# The disconnected: COVID-19 and disparities in access to quality broadband for higher education students

**DOI:** 10.1186/s41239-021-00262-1

**Published:** 2021-05-21

**Authors:** John Cullinan, Darragh Flannery, Jason Harold, Seán Lyons, Dónal Palcic

**Affiliations:** 1 JE Cairnes School of Business & Economics, National University of Ireland Galway, Galway, Ireland; 2 Department of Economics, Kemmy Business School, University of Limerick, Limerick, Ireland; 3 Dublin and Department of Economics, Economic and Social Research Institute, Trinity College Dublin, Dublin, Ireland

**Keywords:** Online learning, Blended delivery, Broadband, Connectivity, COVID-19

## Abstract

The COVID-19 pandemic forced many higher education institutions (HEIs) across the world to cancel face-to-face teaching, close campus facilities, and displace staff and students to work and learn from home. Given the persistent nature of the pandemic, many HEIs have continued to deliver courses online and/or use a blended learning approach. However, there are concerns around differences in student access to digital learning resources while at home, including high quality broadband connectivity. This is important, since variation in connectivity may impact the type of online/blended model that faculty can deliver or constrain student engagement with online content. In this context, this paper combines national data on the domiciles of students enrolled in Irish HEIs with detailed spatial data on broadband coverage to estimate the number of higher education students ‘at risk’ of poor access to high quality internet connectivity. Overall it finds that one-in-six students come from areas with poor broadband coverage, with large disparities by geography and by HEI. It also finds that students from the poorest broadband coverage areas are more likely to be socioeconomically disadvantaged. As a result, this paper recommends that HEIs use their detailed registration data to help identify and support at-risk students. In particular, the results suggest that some HEIs may need to prioritise access to campus facilities and services to less well-off students living in poor broadband coverage areas.

**Supplementary Information:**

The online version contains supplementary material available at 10.1186/s41239-021-00262-1.

## Introduction

In early 2020, the COVID-19 pandemic forced many higher education institutions (HEIs) across the world to cancel face-to-face teaching, close campus facilities, and displace staff and students to work and learn from home. For example, the European University Association (EUA) estimated that 90% of HEIs in Europe ‘went online’ at this time, for all or most of their classes (Gaebel, 2020). Given the persistent nature of the pandemic, and the potential threat of further waves of the virus, many HEIs decided to continue to deliver courses online and/or use a blended learning approach. Evidence from the United States (US) suggests that the majority of colleges have adopted this approach (Staff, 2020), while a similar situation exists in numerous other countries, including the United Kingdom (UK), Australia, and Ireland (Bothwell, 2020; Davies, 2020; McGuire, 2020). While these modes of delivery have existed within the higher education sector for a number of years, the scale of such change is unprecedented and raises a number of important issues.

One such issue is the potential difference in access to digital learning resources for students that reside at home, rather than on or near campus, in an online delivery context (Raes et al., 2019). Such a divide may be driven by a range of factors, including gaps in access to appropriate equipment, such as a laptop or desktop personal computer (PC), a suitable home environment to learn/study in, or the digital literacy skills required to engage with online learning (Silva et al., 2018). Furthermore, differences in the quality of broadband connectivity^1^ for students living at home, as opposed to on campus, is likely also an important consideration in this potential divide (Rasheed et al., 2020). Given the catchment areas of many HEIs cover both urban and rural areas, variation in connectivity may impact the type of online/blended model that staff can deliver, or constrain certain groups of students from fully engaging with online-based content. Within this context, this paper considers college students in Ireland at risk of poor access to high quality internet connectivity due to poor broadband coverage.

With the potential to decrease temporal and spatial constraints relative to traditional higher education offerings, the number of students enrolled in online learning in higher education globally has grown significantly in recent years (Panigrahi et al. 2018). Nonetheless, prior to the pandemic, face-to-face delivery constituted the vast majority of student contact time. Despite this, there were still concerns around broadband connectivity. For example, in the US, Gonzales et al. (2018) estimated that 20% of college students had difficulty maintaining access to technology, including internet connectivity. With the sudden move to emergency online delivery and the widespread closure of campus facilities, the issue has come into sharp focus.

Despite this move, there is limited evidence regarding the impact of differences in broadband access or speeds on learning outcomes in online education at a large scale. Two notable exceptions are Sanchis-Guarner et al. (2021) and Dettling et al. (2018). The former uses data on test scores of 14-year-olds in the UK and finds that increasing broadband speed by 1 Mbps increases test scores by 1.37 percentile ranks, while the latter uses US data to show that students with broadband access in their postal codes perform better on the SAT and apply to a larger set of colleges. To the authors’ knowledge, similar studies in a higher education setting do not exist. However, studies such as Skinner (2019), Rasheed et al. (2020), Raes et al. (2019), and Zydney et al. (2019) all highlight the technological challenges, such as access to high-speed broadband, that can impact on student and teacher engagement with online education, particularly with synchronous-based material.

Given the important role that student engagement likely plays in academic success and student satisfaction, particularly for first year students and in online learning environments (Kahu, 2013; Kahu et al., 2020; Paulsen & McCormick, 2020), this raises the issue of potential differences in the quality or type of delivery students may receive in the current context as a result of unequal broadband access. Furthermore, using survey data from 78 centres for teaching and learning across 23 countries in Spring 2020, Naffi et al. (2020) identified bandwidth issues as problematic for students in certain aspects of their learning experience, such as sharing files or synchronous classes.

In addition to these studies, student survey data from the UK indicated that 7% of students reported having insufficient access to the internet, a figure that rises to 12% for those from lower socioeconomic households (Montacute & Holt-White, 2020). A separate survey found that 56% said they lacked access to appropriate online course materials, with 9% “severely” impacted (Office for Students, 2020). In Ireland, the context for this paper, based upon their experience of online learning in March 2020, 21% of third-level students indicated that access to reliable Wi-Fi was a key requirement to help improve their learning experience going forward (Union of Students in Ireland, 2020).^2^ Overall, these recent student surveys, along with the previous academic research, help motivate the research questions.

### Research questions

From both a policy and HEI management perspective, it is important to understand variation in the quality of home broadband connectivity and to identify groups of students that may be at risk in terms of a pedagogical digital divide. To do so, this paper uses geographic information systems (GIS) techniques to examine national data on the domiciles of students enrolled in Irish HEIs. It combines this information with spatial data on broadband quality from Ireland’s National Broadband Plan (NBP), which allow the number of college students ‘at risk’ of poor access to high quality internet connectivity as a result of coverage issues to be estimated. In considering these ‘disconnected’ students, the paper examines disparities by geography, HEI, and socioeconomic background.

The specific research questions (RQs) that are addressed in this paper are as follows:

- RQ1: How many and what proportion of college students come from areas with poor broadband coverage and are therefore at risk of poor access to high-speed broadband?
- RQ2: Are there significant differences in the proportions of college students from poor broadband coverage areas by geography and by HEI?
- RQ3: Are college students from lower socioeconomic backgrounds more likely to come from poor broadband coverage areas?

In addressing these research questions, the paper is structured as follows: “Literature” section sets out the relevant extant literature, “Institutional and policy context” section describes the context for the study in more detail, “Materials and methods” presents the data and methods, while “Results” section discusses the main empirical results. “Discussion and implications” section summarises the implications of the results and findings, “Conclusion” section concludes, while “Limitations and future research directions” section discusses limitations and future research directions.

## Literature

Due to the recent nature of the crisis caused by the COVID-19 pandemic, there are few empirical studies that examine the impact of broadband coverage on access to online education, at any level of education. One notable exception is Bacher-Hicks et al. (2021), which provides stark evidence of the education digital divide in the US during the COVID-19 lockdown period. Using high-frequency Google search intensity data for online learning resources across 210 different regions, the study shows that areas of the country with higher income levels, better internet coverage, and fewer rural schools saw significantly larger increases in search intensity relative to less advantaged areas. It stresses the importance of additional support for students in low socioeconomic status (SES) areas and rural communities if inequalities in access to, and engagement with, online learning resources are to be reduced (Bacher-Hicks et al. 2021).

In Ireland, a recent study by Mohan et al. (2020) on the impact of the COVID-19 pandemic on second-level education finds that almost half of schools surveyed reported issues with a lack of access to high-speed broadband and/or a lack of access to appropriate digital devices for their students. This figure increases to approximately 58% for disadvantaged schools and schools in catchment areas characterised by lower than median household incomes (Mohan et al., 2020). The survey also finds a significant digital divide in relation to the use of live online video classes. For example, in schools located in areas of lower quality broadband coverage, 62% reported delivering all or most classes live online, compared to 90% of schools located in areas with good broadband coverage. Moreover, in schools located in areas characterised by lower incomes, just under half reported delivering all or most classes live online, compared to almost two-thirds of schools located in higher income areas (Mohan et al., 2020).

Further empirical studies that examine the relationship between the quality of broadband coverage and access to online education during the current pandemic have yet to be published. However, there are a number of pre-pandemic studies that focus on important and related issues, such as: (1) access to quality broadband and appropriate equipment for students; (2) digital inequality driven by socioeconomic factors; and (3) the impact of online learning on student outcomes. While not directly comparable to the research question within this paper, they nonetheless provide important context.

For example, Raes et al. (2019) and Rasheed et al. (2020) both provide systematic reviews of synchronous hybrid learning and the online aspect of blended learning respectively. Raes et al. (2019) suggest grounds for cautious optimism about synchronous hybrid learning in creating an engaging learning environment relative to fully online, but also acknowledge the technological challenges, such as connectivity issues, that may present in such an environment. Rasheed et al. (2020) also highlight the issue of quality broadband (under the heading of technological sufficiency challenges) as one of the main student challenges, but also a potential challenge for staff in a blended learning environment using video content.

In another study, Skinner (2019) uses data from the US national broadband plan to examine the relationship between access to high-speed broadband and the number of students at public universities and community colleges who opt to take some of their courses online. It finds that increases in broadband speed at the lower end of the speed spectrum are positively associated with the number of students who take some of their courses online and emphasises the importance of considering broadband speed in improving the access of students to courses with online content.

While the digital divide in terms of broadband connectivity is obviously important, there can also be gaps in terms of access to appropriate equipment, such as a laptop or desktop PC, the right environment to work in from home, and the digital literacy skills required to engage with online learning. For example, students from lower SES families are less likely to have access to broadband, less likely to have access to a computer, and less likely to have an appropriate learning environment in their home, compared to students from higher SES families (Lamb et al., 2020; Silva et al., 2018). They are also more likely to have relatively weaker information and communications technology (ICT) skills, as well as the capacity for working independently with ICT (Lee, 2017; Ortagus, 2017; Stich & Reeves, 2017).

With regard to broadband uptake, Silva et al. (2018) used census block level fixed broadband availability and broadband adoption data, along with various demographic and socioeconomic variables, to examine the determinants of broadband adoption in the US. The study finds that the broadband availability rate is the most significant factor affecting broadband adoption rates in non-metropolitan areas, while household income and educational attainment play a more significant role in metropolitan areas. Interestingly, it also highlights the importance of shifting the focus of future research away from broadband availability and adoption, towards considering the stability and speed of broadband connections in different geographical areas (Silva et al., 2018). This is particularly important in the context of this paper, since it is not just the availability of broadband that matters for students. Rather, it is broadband quality or performance, defined in terms of upload/download speeds and latency, which is a critical factor for learners engaging with many of the applications and technologies used for synchronous sessions.

In terms of the extant empirical literature on the impact of online learning on student engagement and educational outcomes, this has generated mixed and contested results (Paulsen & McCormick, 2020). For example, Xu and Jaggars (2013a) use a large administrative dataset from a state-wide system of 34 community and technical colleges in Washington State in the US to estimate the impact of online versus face-to-face delivery on academic performance. The study, using various approaches and model specifications, indicates that online delivery has a significant negative impact on both course grade and course persistence. On the other hand, Figlio et al. (2013) provide experimental estimates of the effects of online versus face-to-face instruction on student learning and find only modest evidence in favour of face-to-face delivery.

While there is no clear-cut consensus as to the efficacy of online learning versus face-to-face delivery, Xu and Jaggars (2013b) argue that the gap between online and face-to-face outcomes may be more significant for less-advantaged cohorts. The authors suggest that gaps in outcomes may therefore be higher for colleges with higher proportions of disadvantaged students and less obvious for institutions that serve more socially advantaged students with better prior academic ability. Farrell and Brunton (2020) also highlight how successful online student engagement is influenced by “a number of psychosocial factors such as peer community, an engaging online teacher, and confidence or self-efficacy and by structural factors such as lifeload and course design”.

While many of the studies discussed in this section relate to the pre-pandemic online education experience of relatively small numbers of students, the findings are nonetheless highly relevant to the current experience of a rapid transition to mass online learning. In particular, they underline the importance of access to high-speed broadband in an online learning environment.

## Institutional and policy context

### Higher education landscape

HEIs in Ireland include universities, technological universities (TUs), institutes of technology (ITs), and colleges of education (CEs), as well as a small number of other public and private colleges. In 2018/19 enrolments totalled 228,503, with the majority of those (186,174; 81%) at undergraduate level (Higher Education Authority, 2020). Of those enrolled, 55% were in the university sector, 40% were in TUs/ITs, with the remaining 5% in other colleges (Higher Education Authority, 2020). Health and humanities courses at honours bachelor degree level are more common in the university sector, while a focus on engineering, construction, and care courses at both ordinary and honours bachelor degrees is more common in TUs and ITs. Compared to universities, TUs and ITs offer more part-time and flexible courses, with a larger proportion of mature and disadvantaged students, while universities offer more postgraduate opportunities (Higher Education Authority, 2019). Flannery and Cullinan (2017) present further details of the Irish higher education sector.

From a spatial perspective, universities and CEs in Ireland tend to be located in larger urban centres, whereas TUs/ITs are more geographically dispersed and smaller in size on average (Additional file 1: Figures A.1 and A.2). A substantial body of research has examined student mobility and enrolment patterns in Ireland.^3^ In general, these studies have found that proximity to a HEI strongly influences where a student enrols and these ‘localised’ patterns of progression to HEIs are likely important in the context of understanding disparities in access to quality broadband services. In terms of financial aid, the Irish State provides maintenance grants to students who meet certain criteria based on parental income levels and geographic distance from their chosen HEI. It is also relevant to note that the most recent equity of access plan in the sector, the *National Plan for Equity of Access to Higher Education 2015–2019* (Higher Education Authority, 2015), while not focusing on potential digital divides specifically, acknowledges the potential role that geographic factors may play in higher education accessibility.

On March 12th 2020 all HEI facilities in Ireland, including libraries, offices, classrooms and labs, were closed to staff and students with the remainder of the spring semester’s teaching delivered remotely. With student accommodation almost entirely vacated, this emergency shift to online teaching and alternative assessment resulted in the vast majority of students learning from home. For the 2020/21 academic year, a fully online or blended learning approach was adopted by all HEIs and, in response, a €15 million funding package was announced to help with online learning to assist students from lower incomes access laptops, tablets, and internet connectivity. The majority of this funding was earmarked for laptop purchases and with little detail on the connectivity issues that it may help address (Department of Education & Skills, 2020).^4^

In addressing the specific responsibilities of HEIs in relation to quality assurance in a blended learning environment, Quality and Qualifications Ireland provide statutory quality guidelines (Quality & Qualifications Ireland, 2018). It is noteworthy that no mention of desirable broadband speeds for instructors or students is made in their report. However, under the heading of support available to students, it is recommended that “requirements for access, bandwidth and any prescribed hardware or software are appropriate and viable, are communicated well in advance to the learners and all requirements are published” (Quality & Qualifications Ireland, 2018). With regard to equality of opportunity, the guidelines also state that procedures in place include “teaching and learning resources for online learning which meet the provider’s specified expectations around equality of opportunity, interactivity and the empowerment of autonomous learning” (Quality & Qualifications Ireland, 2018). While such guidelines were written pre-COVID-19 when blended learning was ‘optional’, they have not been changed or updated at a time when many HEIs have pivoted towards a fully online or blended model of delivery and are highly relevant in the context of understanding access to high quality broadband in Ireland.

### Broadband connectivity in Ireland

The digital divide between urban and rural areas in terms of access to high-speed broadband services in Ireland has long been recognised (Commission for Communications Regulation, 2009).^5^ However, Ireland has generally lagged considerably behind its European peers in terms of implementing policy measures to address the issue (Palcic & Reeves, 2011). Ireland’s National Broadband Plan (NBP) was first published in August 2012 and set a target of a minimum download speed of 30Mbps for all households ahead of the EU’s target of 2020 for such speeds. However, a contract notice for the NBP was not issued until December 2015, with the signing of a contract with the preferred bidder delayed until November 2019 after a highly controversial procurement process.

The intervention area for the NBP includes rural areas that currently do not have access to high-speed broadband services and where commercial operators have no plans to deploy such services. Work commenced on the rollout of the NBP in 2020 and it is estimated that the plan will be fully delivered by 2026, with 40% of premises ‘passed’ by the end of the third year of deployment (Department of Communications, Climate Action and the Environment, 2019). As part of the plan, approximately 300 broadband connection points (WiFi hotspots) were to be deployed across the country by the end of 2020, so that those in rural communities can access high-speed broadband services in specific locations in advance of the full deployment of the NBP network.

**Fig. 1:**
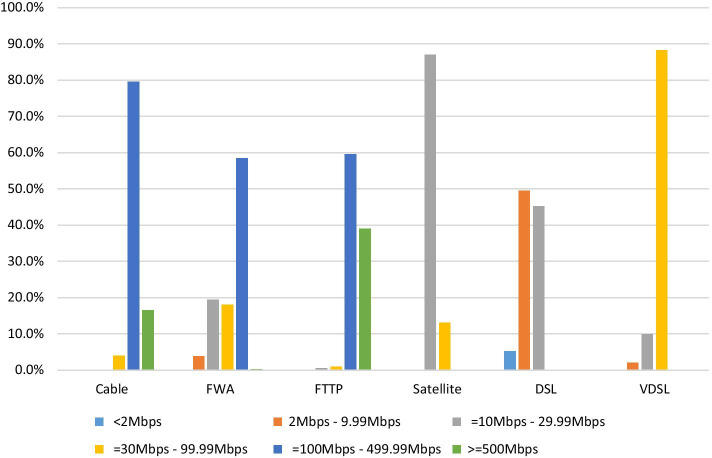
Advertised broadband download speeds by fixed platform, Q3 2020. *FWA*  fixed wireless access, *FTTP* fibre to the premises, *DSL* digital subscriber line, *VDSL* very-high-bit-rate digital subscriber line. Source ComReg (2020a)

To gain a better insight into the broadband services currently available across various platforms in Ireland, data provided by Commission for Communications Regulation (2020a) shows that, at the end of September 2020, total broadband subscriptions stood at 1.83 million, with fixed broadband subscriptions accounting for 82.3% of this total and mobile broadband subscriptions accounting for the remainder.^6^ However, the vast majority of higher speed FTTP (fibre to the premises), cable and VDSL (very-high-bit-rate digital subscriber line) fixed broadband services are only available in urban areas, with basic DSL (digital subscriber line), FWA (fixed wireless access), mobile broadband, and satellite services predominantly used in rural areas. Figure 1 shows the advertised headline speeds across each fixed broadband platform and provides some insight into the digital divide in terms of broadband performance between urban and rural areas. Basic DSL services have advertised download speeds of less than 30 Mbps, with over half of such connections in less than 10 Mbps range. Approximately 40% of FWA services have download speeds of less than 30 Mbps, while the vast majority of satellite services have download speeds of less than 30 Mbps. In contrast, VDSL, cable, and FTTP services offer download speeds well in excess of 30 Mbps.

In terms of mobile broadband services, the average download speeds for 3G and 4G mobile broadband connections are not available outside of cities and major transport routes. While the 3G and 4G coverage maps of each of the main mobile operators in Ireland show that the vast majority of the country is covered, the quality and stability of this coverage can vary widely, particularly in rural areas where coverage can be extremely poor. Various mobile consumer experience surveys conducted by the Commission for Communications Regulation have highlighted the fact that indoor mobile reception has a far higher incidence of experiencing service issues due to modern building materials (Commission for Communications Regulation, 2017a, 2019). This issue is amplified in rural areas where the Commission for Communications Regulation surveys reveal that rural mobile consumers experience the highest rates of service issues regardless of location within or outside the home (Commission for Communications Regulation, 2018). In recent years, the Commission for Communications Regulation have also expressed concern at the increase in the number of illegal roof aerials and mobile booster devices in rural areas, which can cause considerable interference to mobile phone spectrum and lead to significant deterioration in mobile reception in areas that already have limited coverage (Commission for Communications Regulation, 2017b).

Outside of the high-speed broadband services provided by FTTP, cable, and most VDSL connections, it is subscribers to DSL, mobile, and FWA services that are more likely to have experienced connectivity issues during the lockdown caused by the pandemic. These issues were caused by higher data volumes on networks due to more people working from home, students at all levels engaging in online learning, and increased download activity in general as people accessed streaming video services or online gaming platforms (New York Times, 2020). Indeed, platforms such as Netflix, YouTube, and Disney were forced to temporarily throttle their video streams across Europe during March and April 2020, in order to limit bandwidth usage and ease pressure on congested networks (Financial Times, 2020).

In Ireland, a Commission for Communications Regulation survey in April 2020 found that over 60% of households increased their broadband usage during the lockdown, with 74% of households indicating that their home broadband connection was adequate for all work activities. However, this percentage fell to 67% for those living in rural areas, with households utilising either a DSL or mobile broadband connection also recording the lowest levels of satisfaction with the adequacy of their connection (Commission for Communications Regulation, 2020b). More recent Commission for Communications Regulation survey data from June 2020 shows that household internet usage has increased further since April 2020, with those in rural areas continuing to have the lowest satisfaction level with the adequacy of their home broadband connection (Commission for Communications Regulation, 2020c). The same survey also highlights how half of all participants indicated that they would be willing to spend more to get a better broadband service, showing the increased reliance on broadband for all households.

While it is very difficult to identify the exact download speed that a student would need to be able to fully engage with all aspects of online learning, particularly synchronous interactive video sessions, it is highly likely that students using basic broadband technologies will be affected most by connection issues. Such issues would be exacerbated if there are multiple internet users in the same household, as well as neighbouring households, where contention and congestion would severely impact available download speeds on technologies such as DSL. With continued uncertainty in relation to the potential for future surges in the pandemic, and the likelihood that many people will continue to have to work and learn remotely in the near future, those with poor fixed or mobile broadband services will continue to be at a major disadvantage relative to households with more stable higher speed services. This digital divide has the potential to create significant inequalities in education at all levels, particularly for students from lower socioeconomic backgrounds, who are more likely to experience issues in relation to access to, and affordability of, broadband and appropriate devices.

## Materials and methods

### Data

The overarching goal of this paper is to assess access to high-speed broadband among college students in Ireland in the context of significantly increased levels of remote learning arising from the COVID-19 pandemic. It also seeks to examine disparities in access by geography, HEI and socioeconomic background. To do so, the analysis combines a variety of spatial data from four main sources.

First, this paper uses unique data on higher education student enrolments for 2017/18 from the Higher Education Authority (HEA).^7^ This data defines an enrolment as a student registered in an Irish HEI and is based on a census of all enrolments undertaken in March 2018. For the academic year 2017/18 there were a total of 223,743 enrolments at Irish HEIs. Additional file 1: Table A.1 presents a breakdown of enrolments by HEI),^8^ though in this analysis data from TCD (16,755 enrolments) are excluded, due to non-reporting of relevant domicile data to the HEA, as are data from UCC (20,024 enrolments), due to likely misreporting of the same domicile data. Enrolments from outside Ireland are also excluded and, overall, this provides data on 167,576 enrolments for the 2017/18 academic year.

The HEA data includes information on student domicile, which is based on the address of permanent residence for 3 of the 5 years prior to initial enrolment. This information is available at electoral division (ED) level, small-scale geographic areas of which there are 3,409 in total. The population and geographic coverage of EDs vary considerably, with a mean overall population of 1,397 (range: 66 to 38,894) and a mean area of 19.6 km^2^ (range: 0.01 to 125.94km^2^). Thus, EDs provide a high level of spatial disaggregation in relation to student domicile. Finally, the HEA data also contains information on undergraduate (143,214; 85%) and postgraduate (24,362; 15%) enrolments, full-time (133,756; 80%) and part-time (33,820; 20%) enrolments, as well as the specific HEI each student attends.

The second dataset used is digital data on high-speed broadband coverage based on a mapping exercise undertaken by the Department of Communications, Climate Action and Environment (DCCAE). As discussed, the NBP is the Government’s plan to deliver high-speed broadband services to all premises in Ireland. As part of the NBP, a comprehensive analysis and mapping process of high-speed broadband availability across the country was undertaken, which involved the development of an interactive map which identifies geographic areas as being either served by the commercial sector or requiring State intervention under the NBP.^9^

The DCCAE High-speed Broadband Map shows where high-speed broadband services are currently available and identifies locations and premises as being either ‘amber’, ‘blue’, or ‘light blue’. Amber areas are the target areas for the State intervention of the NBP i.e., areas in which there is currently no high-speed broadband. Blue areas are areas where commercial operators are delivering or have indicated plans to deliver high-speed broadband services, while light blue areas are areas where a commercial operator has committed to rollout high-speed broadband to 300,000 premises. Overall, of the 2,391,559 premises in the country as of Q3 2019, there were 537,595 (22.5%) premises in the intervention area (amber), 1,838,932 (76.9%) covered by commercial operators (blue), and 15,032 (0.6%) premises to be covered by planned commercial rural deployment.

The third dataset is the An Post GeoDirectory, a database of all residential and commercial buildings in Ireland, each matched to a unique postal address and geocoded to within a single square metre. A geocode is a code or reference number which pinpoints a specific location and the references or (X,Y) coordinates in GeoDirectory are GIS compatible. This means that the database can be easily mapped and analysed in conjunction with other spatial data e.g. attribute data on EDs or broadband coverage. In the analysis GeoDirectory data from 2018 is used.

Fourth, and finally, this paper also uses Census of Population-based data from 2016 to develop a profile of EDs with poor access to high-speed broadband. This includes an area-level deprivation index score (Haase & Pratschke, 2017), as well as ED-level small area population statistics (SAPS) data on median household income and PC ownership (CSO, 2020). The deprivation index is a measure of relative socioeconomic position based on the area of each home address. Scores range from -35.7 to + 16.8, with a mean of -5.0, and higher scores indicate greater affluence. Median household income is measured in Euros, while PC ownership is measured by the proportion of households in an ED with a computer.

### Methods

**Fig. 2:**
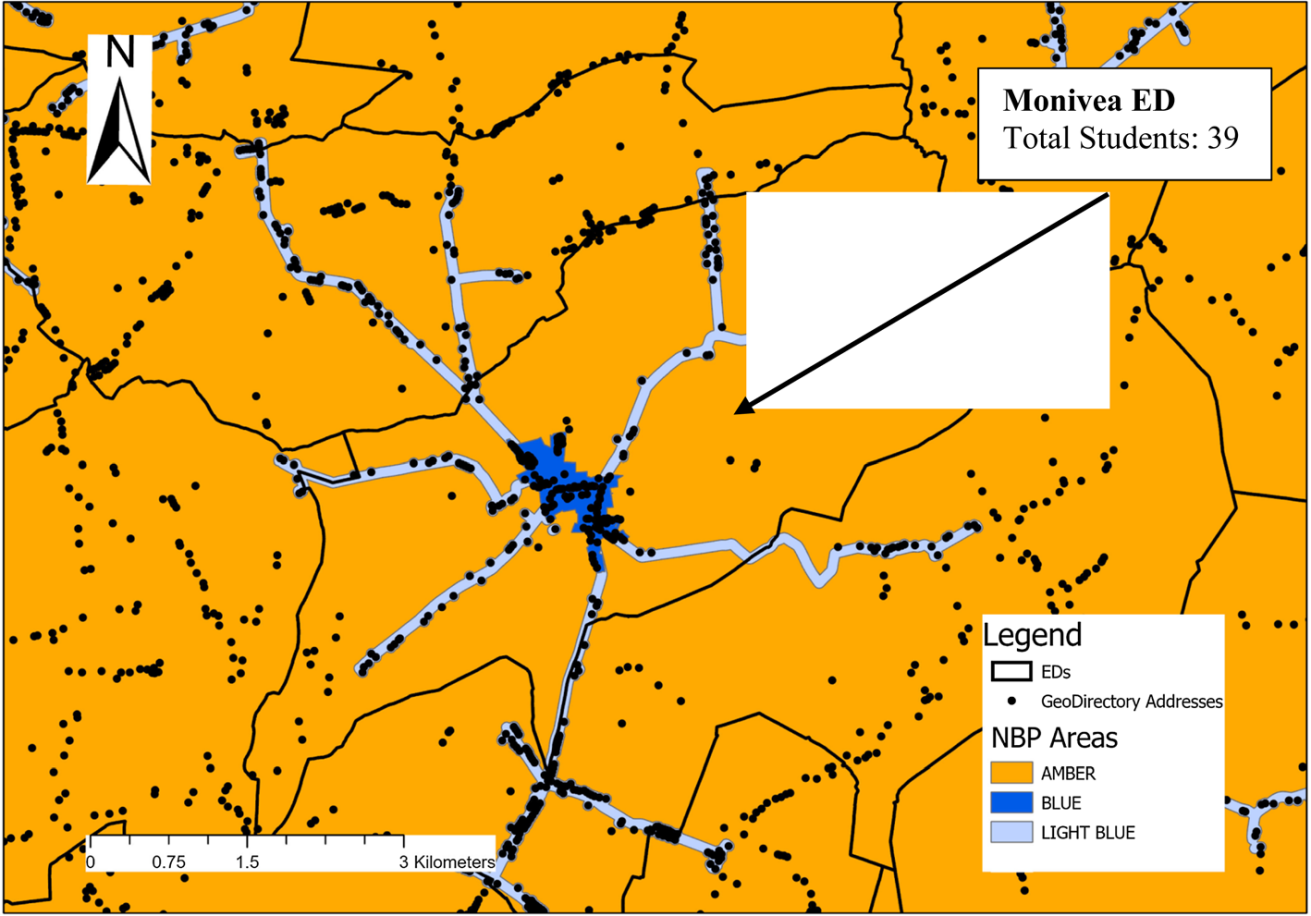
Geographic information systems approach. Source: Analysis of data from HEA, GeoDirectory, and National Broadband Plan

To undertake the analysis and address the three RQs, GIS methods are used to (i) create ‘at-risk’ measures of poor access to high-speed broadband in the domicile areas of college students and, (ii) develop a socioeconomic profile of these at-risk areas. A GIS is an interactive computer program capable of assembling, storing, analysing, and displaying information that has been identified by location and, at its most basic level, a GIS application can be thought of as a computerised map. For example, to begin, the HEA enrolment data is first mapped at ED level. This allows the total number of college students with a domicile in each ED to be both stored and visually inspected, as well as the numbers of students attending each individual HEI with a domicile in that ED. For example, Fig. 2 shows a total of 39 students with a domicile in Monivea ED and the data disaggregates this total by HEI attended.

At a more sophisticated level, a GIS can also help analyse multiple and complex layers of data, matching them to a specific point, locality, or area. The second step involves ‘overlaying’^10^ the DCCAE NBP map with the geocoded data on residential addresses from GeoDirectory, as illustrated in Fig. 2. More specifically, using a ‘spatial join’,^11^ it is possible to infer, for every residential address in Ireland, whether it has access to high-speed broadband. This is defined on the basis of whether the address is located in an amber or light blue area (i.e., no current high-speed broadband availability) or a blue area (i.e., currently has high-speed broadband availability)—Fig. 2. It is then straightforward to calculate the proportion of residential addresses in each ED with high quality broadband access and, for different cut-offs or measures of areas at risk of poor access, to calculate the numbers of college students residing in these areas.

To do this, four measures or levels of at-risk areas are defined in terms of high-speed broadband coverage/availability. This approach follows that of Mohan et al. (2020), which in their study of second-level education in Ireland during COVID-19, created an indicator of ‘low broadband availability’ for secondary school catchment areas where high-speed broadband was available to fewer than 90% of residences, according to the NBP map. In defining at-risk areas in this paper, a lower set of thresholds are adopted, though the sensitivity of the results to less stringent assumptions are tested. In the main analysis, EDs are first classified at an overall level as having poor broadband coverage if high-speed broadband services are available at fewer than 50% of addresses. These EDs are then disaggregated into one of four mutually exclusive at-risk categories i.e. either (i) ‘low coverage’, where high-speed broadband is available at between 25 and 50% of residential addresses, (ii) ‘very low coverage’, where availability is between 10 and –25% of addresses, (iii) ‘minimal coverage’, where availability is between 0 and 10% of addresses, and (iv) ‘no coverage’, where broadband is available at 0% of residential addresses. This allows for the estimation of the numbers and proportions of college students living in areas with poor broadband coverage and who are therefore at risk of poor access to high quality internet connectivity according to a variety of at-risk measures (RQ1), and to consider differences in access by geography, HEI, and area-level characteristics (RQ2 and RQ3). In considering area-level characteristics, tests of statistical differences in means between EDs defined as having poor broadband and those defined as not are undertaken. To do so, standard two-sample t-tests assuming unequal variances are used.

## Results

To begin, Table 1 presents a breakdown of total enrolments according to the at-risk measures and helps answer RQ1 i.e. how many and what proportion of college students come from areas with poor broadband coverage and are therefore at risk of poor access to high-speed broadband? Overall, of the 167,576 students in the data, 16,462 (9.8%) had domiciles in EDs with low broadband coverage, 6008 (3.6%) in very low coverage EDs, 2801 (1.7%) in minimal coverage EDs, and 2,598 (1.6%) in EDs with no high-speed broadband coverage. Overall this implies that 16.6% of students (27,869) in the data were at risk of poor broadband access during the COVID-19 lockdown, assuming a 50% ED coverage threshold.^12^ Table 2 presents a similar analysis for undergraduate and postgraduate students, as well as for full-time and part-time students. It shows that undergraduate and full-time students are more likely to face broadband access issues relative to postgraduate and part-time students respectively.

**Table 1:** Number and proportion of higher education student enrolments at risk of poor access to high speed broadband

ED at-risk measure	Enrolments	Enrolments (%)
Low coverage EDs	16,462	9.8%
Very low coverage EDs	6008	3.6%
Minimal coverage EDs	2801	1.7%
No coverage EDs	2598	1.6%
All poor coverage (at-risk) EDs	27,869	16.6%
All EDs	167,576	

Low coverage denotes between 25 and 50% of residential addresses in ED have access to high speed broadband services as per National Broadband Plan map, very low coverage denotes between 10 and 25%, minimal coverage denotes between 0 and 10%, and no coverage denotes no residential addresses in ED have access to high speed broadband services. Poor coverage is defined as fewer than 50% of residential addresses in an ED having access to high speed broadband services

Source: Analysis of data from HEA, GeoDirectory, and NBP

**Table 2:** Number and proportion of undergraduate, postgraduate, full-time, and part-time student enrolments at risk of poor access to high speed broadband

ED at-risk measure	UG	UG (%)	PG	PG (%)	FT	FT (%)	PT	PT (%)
Low coverage EDs	14,414	10.1%	2,048	8.4%	13,729	10.3%	2733	8.1%
Very low coverage EDs	5286	3.7%	722	3.0%	5029	3.8%	979	2.9%
Minimal coverage EDs	2468	1.7%	333	1.4%	2340	1.7%	461	1.4%
No coverage EDs	2330	1.6%	268	1.1%	2245	1.7%	353	1.0%
All poor coverage (at-risk) EDs	24,498	17.1%	3371	13.8%	23,343	17.5%	4526	13.4%
all EDs	143,214		24,362		133,756		33,820	

Low coverage denotes between 25 and 50% of residential addresses in ED have access to high speed broadband services as per National Broadband Plan map, very low coverage denotes between 10 and 25%, minimal coverage denotes between 0 and 10%, and no coverage denotes no residential addresses in ED have access to high speed broadband services. Poor coverage is defined as fewer than 50% of residential addresses in an ED having access to high speed broadband services. UG denotes undergraduate, PG denotes postgraduate, FT denotes full-time, and PT denotes part-time

Source: Analysis of data from HEA, GeoDirectory, and NBP

**Fig. 3:**
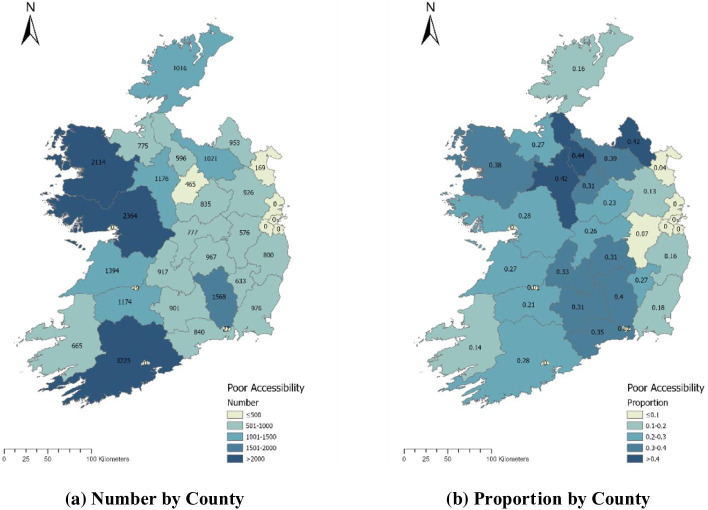
Number and proportion of enrolments from at-risk electoral divisions by county. Source: Analysis of data from HEA, GeoDirectory, and National Broadband Plan

While Tables 1 and 2 give a good sense of the overall picture at a national level, it is also important to consider regional or geographical differences in access to high-speed broadband for college students – this addresses part of RQ2 i.e. are there significant differences in the proportions of college students from poor broadband coverage areas by geography and by HEI? To this end, Fig. 3 presents a breakdown by county of both the number and proportion of at-risk enrolments i.e. students with a domicile in EDs with below 50% coverage. It shows the highest absolute numbers of students without good broadband access are located mainly in the west and south-west of the country. In proportionate terms, however, it is counties in the north midlands and border area that are most disadvantaged in terms of access. The former result is in part driven by the high numbers of students from those counties, while the latter is a function of the generally poor broadband coverage in those areas. One caveat here is that, as mentioned, the data do not include enrolments at TCD or UCC, and this will likely distort the regional analysis to some extent. For example, the numbers of students in the south-west of the country are likely to be understated, given this is the catchment area of UCC. Nonetheless, they do suggest a strong regional dimension to broadband access issues.

**Fig. 4:**
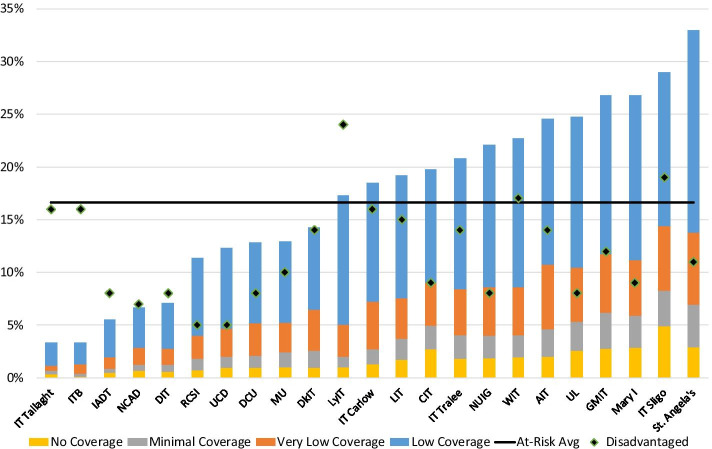
Proportion of enrolments from at-risk electoral divisions by HEI. Source: Analysis of data from HEA, GeoDirectory, and National Broadband Plan

As mentioned previously, there is extensive evidence of localised patterns of progression to HEIs in Ireland (Cullinan & Duggan, 2016; Cullinan & Halpin, 2017). In other words, students are much more likely to attend a HEI that is closer to their home than one farther away. Given this, along with the regional disparities illustrated in Fig. 2, significant differences in domicile access to broadband by HEI might also be expected, as per RQ2. To investigate this, the proportions of enrolments at risk of poor broadband access by HEI were calculated. Figure 4 shows that, perhaps not unsurprisingly, there is considerable variation across HEIs in the measures that are used.

For example, starting with HEIs with good broadband access, 2.2% of students at IT Tallaght are from low coverage EDs, 0.5% from very low coverage EDs, 0.3% from minimal coverage EDs, and 0.3% from EDs with zero coverage. Thus, overall, only 3.3% of IT Tallaght students are classified as having poor access to high quality broadband as per the definition used here. However, at the other end of the spectrum, this is in stark contrast to St Angela’s College, where 19.2% of students are from low coverage EDs, 6.8% from very low coverage EDs, 4.0% from minimal coverage EDs, and 2.9% from EDs with zero coverage. Therefore, overall, 33.0% of students enrolled at St Angela’s College are classified as at risk.

The findings from the HEI analysis are consistent with the county-level analysis of enrolment numbers. The eight HEIs with the lowest proportion of at-risk students are located in Dublin, where broadband quality is high. HEIs with the highest proportions of at-risk students tend to be located in the west or midlands. Again, this is a function of the catchment areas of individual HEIs, as well as the unequal spatial distribution of high-quality broadband in Ireland. However, an important point to note here in the context of differences across HEIs is that broadband access is only one possible cause of the digital divide and some HEIs that have good access may face alternative challenges. For example, Fig. 4 also presents the proportion of socioeconomically disadvantaged students by HEI. Although not a primary focus of this paper, the data indicates that there is considerable variation in this measure across HEIs and this could be associated with other issues around digital learning resources for both students and HEIs e.g. affordability of, and access to, appropriate equipment, suitable home learning environments, and/or digital literacy skills.

The final piece of analysis involves considering differences in the socioeconomic profile of at-risk EDs in order to examine RQ3 i.e. are college students from lower socioeconomic backgrounds more likely to come from poor broadband coverage areas? In order to do this, this study examined data on area-level deprivation, median household income, as well as PC ownership by ED, and the results are presented in Table 3. It also presents tests for statistical differences in the means of these variables between at-risk EDs and non-poor coverage EDs using two-sample t-tests. Overall, the results show that EDs with the poorest broadband coverage tend to be the most deprived and have the lowest median household income, though only the differences between no coverage EDs and non-poor coverage EDs are statistically significant. While there is little difference (either practically or statistically) in these socioeconomic measures overall for poor coverage areas when compared to non-poor coverage areas, it is notable that there is a gradient in both ED-level deprivation and median income as broadband coverage decreases. Thus, overall the evidence suggests that students from areas with the lowest levels of broadband coverage are more likely to be socioeconomically disadvantaged on average.

**Table 3:** Socioeconomic profile of at-risk electoral divisions

ED at-risk measure	ED-level deprivation	Median household income (€)	PC ownership (proportion)
Low coverage EDs	− 4.70 (4.96)	45,245 (9,531)	0.66 (0.07)
Very low coverage EDs	− 4.90 (4.89)	44,639 (9,096)	0.65 (0.07)**
Minimal coverage EDs	− 5.31 (4.83)	43,890 (9,333)	0.64 (0.07)***
No coverage EDs	− 6.30 (5.58)***	41,207 (10,019)***	0.63 (0.09)***
All poor coverage (at-risk) EDs	− 5.10 (5.06)	44,241 (9,588)	0.65 (0.08)
All non-poor coverage EDs	− 4.97 (7.39)	44,693 (12,835)	0.67 (0.09)
All EDs	− 5.03 (6.39)	44,477 (11,403)	0.66 (0.09)

Low coverage denotes between 25 and 50% of residential addresses in ED have access to high speed broadband services as per National Broadband Plan map, very low coverage denotes between 10 and 25%, minimal coverage denotes between 0 and 10%, and no coverage denotes no residential addresses in ED have access to high speed broadband services. Poor coverage is defined as fewer than 50% of residential addresses in an ED having access to high speed broadband services. The table also presents results from two-sample t-tests with unequal variances of the difference in means between at-risk EDs and non-poor coverage EDs. ***p < 0.01, **p < 0.05

Source: Analysis of data from HEA, GeoDirectory, NBP, and CSO

In addition to these two measures, data on PC ownership at ED level was also examined. Here statistically significant differences are found for very low, minimal, and no coverage EDs when compared to non-poor coverage EDs. Again, a gradient in PC ownership is evident, with lower ownership as broadband coverage decreases. This provides some evidence that students with poorer access to broadband services may also be disadvantaged in terms of access to computers for study purposes.

## Discussion and implications

The persistent nature of the COVID-19 pandemic has forced many HEIs to move to mass online/blended learning. This raises concerns around differences in student access to digital learning resources while at home, including access to high-speed broadband services. This is because variation in the quality of broadband access may impact the type of online/blended model that staff can deliver and constrain how students can engage with online content. In this context, this study uses national data on higher education enrolments and broadband coverage to address three research questions relating to the numbers of students at risk of poor access to high-speed broadband and the variation in these numbers by geography, HEI, and socioeconomic background. Overall, the results suggest that almost 17% of higher education students in Ireland come from areas with poor broadband coverage, a figure that is consistent with the proportion of students that indicated access to reliable Wi-Fi was problematic in Spring 2020 (Union of Students in Ireland, 2020), as well as with data from the UK (Office for Students, 2020). (RQ1). They also show considerable variation by geography, as well as by HEI (RQ2). For example, more than a quarter of students in a number of HEIs come from areas with limited broadband coverage. Furthermore, the analysis presented shows that students facing the greatest constraints in terms of broadband coverage are more likely to be socioeconomically disadvantaged (RQ3).

An important implication of the findings is that some HEIs may have to significantly adjust their online delivery methods due to the considerable technological constraints that many students face. In addition, the findings also imply that different groups of students within each HEI may require different offerings, or have different capabilities to access blended/online content. As these constraints are largely based upon spatial factors, it is suggested that HEIs pay specific attention to the geographic pattern of their enrolments and consider tailoring their delivery or services to acknowledge these potential constraints. In this context, it may also be pertinent to ensure teaching staff attempt to gauge the connectivity of their students before deciding on a delivery strategy, if feasible.

On a related point, it should be noted that the analysis in this paper is based on data at ED level rather than at the specific household level, which would be preferable. Furthermore, a relatively conservative measure of which EDs have poor broadband coverage has been used and, as a result, the results may underestimate the true scale of at-risk students. Therefore, it is recommended that HEIs and government agencies use their more in-depth enrolment data to help more precisely identify individual at-risk students. Such an approach would allow HEIs to better develop policies and supports for students that face such connectivity issues. For example, HEIs could prioritise access to campus facilities for those from areas with poor coverage that are living at home to help ensure an effective and equal learning experience for all students. This could also possibly extend to offering subsidised on-campus accommodation for disconnected students from lower income backgrounds.

## Conclusion

The issues raised in this study are not unique to Ireland, with problems relating to digital divides prevalent in the majority of developed and developing countries. This study points to potential connectivity issues for different groups of students in different HEIs. This may be an issue to varying degrees across different countries but is clearly worth examining since it highlights the need for HEIs to consider the geographic distribution of their students in designing appropriate policy and supports if moving towards mass online/blended delivery methods in response to COVID-19-related restrictions.

## Limitations and future research directions

In terms of the analysis, a number of caveats should be borne in mind. First, in using the NBP intervention area mapping data, it is possible that some students in at-risk EDs have basic DSL or mobile broadband connections with download speeds that provide adequate support for most online learning applications. Nevertheless, survey data published by Commission for Communications Regulation (2020a, 2020b) highlights that consumers with DSL or mobile broadband connections, as well as those in rural areas, had the lowest satisfaction rates in relation to the adequacy of their connection since the pandemic commenced. Second, data on in-home capacity issues such as WiFi quality or the number of people sharing home networks was not available. Such issues can impact the download speeds available within the home regardless of whether a household has access to a high-speed broadband technology or not. Third, it should be noted that there are some temporal differences across the four main data sources used in this paper. However, it is unlikely that the spatial distributions of the measures considered (i.e. higher education student domiciles, broadband availability, residential addresses, and census-based variables) will have changed considerably since the timing of their respective data. In addition, the underlying data is unlikely to have been directly affected by the pandemic. Finally, this paper has not considered whether some courses might require more bandwidth or lower latency broadband services than others, depending on the online learning applications being utilised. This is because course-level domicile data was not available.

With regard to future related research, it is suggested that HEIs or relevant teaching staff monitor the performance of students from areas of poor connectivity to evaluate variation in student engagement or performance relative to their better connected peers. For example, the results show that many ITs in Ireland have significant numbers of students with domiciles in poor broadband areas. Given that, relative to universities, progression beyond first year is a significant issue for ITs (McCoy & Byrne, 2017), this may be an issue that could be exacerbated by poor connectivity. Monitoring such issues could help inform the need for additional supports or services for these students.

## Supplementary Information

**Additional file 1.** Additional HEI and enrolment information.

## Data Availability

The data used in this paper are available on request from the Higher Education Authority in Ireland (student domicile data), the Department of the Environment, Climate and Communications (National Broadband Map), and An Post (GeoDirectory).

## Footnotes

1 In the US, the Federal Communications Commission defines broadband as internet speeds of at least 25 megabits per second (Mbps) for downloads and 3 Mbps for uploads (Federal Communications Commission, 2018), while download speeds of less than 30 Mbps are defined as basic broadband in the EU.

2 It is also important to note that in the Irish National Digital Experience (INDEX) survey (National Forum for the Enhancement of Teaching and Learning in Higher Education, 2020), 77% of students sampled indicated they had access to reliable Wi-Fi. However, this survey was conducted in the autumn of 2019, prior to the pandemic, and focused on access on-campus, rather than off-campus. As a result, it is not of immediate relevance to the analysis in this paper.

3 Examples include Walsh et al. (2015), Cullinan and Duggan (2016), Cullinan and Halpin (2017), and Walsh et al. (2017).

4 This package also included the further education sector in Ireland, which is not within the focus of this paper.

5 Commission for Communications Regulation (ComReg) is the Irish communications regulator.

6 Figure B.1 in Additional file 1: Appendix B provides a breakdown of fixed broadband subscription proportions by platform as of Q3 2020.

7 Spatially disaggregated enrolment data for the 2018/19 and 2019/20 academic years are not yet available.

8 This compares to a total of 228,503 enrolments in 2018/19 as per Sect. 3.

9 The DCCAE NBP map was first issued in November 2014 and, following requests for supplementary information from operators, was re-issued in December 2015. In April 2017, DCCAE published an updated ‘High-speed Broadband Map’ that took account of commercial operator plans and new developments and, since then, the map is updated on a quarterly basis.

10 Overlay analysis is a GIS operation that integrates spatial data with attribute data by combining information from one GIS ‘layer’ (e.g. the NBP map) with another GIS layer (e.g. GeoDirectory addresses) to derive or infer an attribute for one of the layers.

11 A spatial join combines the attributes of two layers based on the spatial location of the features in the layers. It does so by appending the attributes of one layer to another within the GIS. It is possible to then use the additional information to perform analysis on the combined data.

12 This proportion increases to 22.4% (37,501 students) if a 60% coverage threshold is assumed, 30.9% (51,783) for a 70% threshold, 39.8% (66,618) for an 80% threshold, and 50.3% of students (84,306) assuming a 90% coverage threshold.

## References

[CR1] Bacher-Hicks, A., Goodman, J., & Mulhern, C. (2021). Inequality in household adaptation to schooling shocks: Covid-induced online learning engagement in real time. *Journal of Public Economics,**193*, 104345 34629567 10.1016/j.jpubeco.2020.104345 PMC8486492

[CR2] Bothwell, E. (2020). UK universities favour blended learning approach for 2020–21. Times Higher Education. https://www.timeshighereducation.com/news/uk-universities-favour-blended-learning-approach-2020-21. Accessed 28 July 2020.

[CR3] Commission for Communications Regulation. (2009). *Next generation broadband in Ireland: promoting the timely and efficient development of high-speed broadband services*. ComReg Document 09/56. Dublin: Commission for Communications Regulation.

[CR4] Commission for Communications Regulation. (2017a). *Mobile consumer experience survey: Summer 2017*. ComReg Document 17/100a. Dublin: Commission for Communications Regulation.

[CR5] Commission for Communications Regulation. (2017b). *Consultation on permitting the general use of mobile phone repeaters.* ComReg Document 17/103. Dublin: Commission for Communications Regulation.

[CR6] Commission for Communications Regulation. (2018). *Permitting the general use of mobile phone repeaters. Response to ComReg consultation document 17/103 and final decision*. ComReg Document 18/58. Dublin: Commission for Communications Regulation.

[CR7] Commission for Communications Regulation. (2019). *Mobile consumer experience: Survey of consumers summer 2019*. ComReg Document 19/101. Dublin: Commission for Communications Regulation.

[CR8] Commission for Communications Regulation. (2020a). *Quarterly key data report. Data as of Q3 2020*. ComReg Document 20/119. Dublin: Commission for Communications Regulation.

[CR9] Commission for Communications Regulation. (2020b). *Impact of COVID-19 on home broadband use in Ireland*. ComReg Document 20/35. Dublin: Commission for Communications Regulation.

[CR10] Commission for Communications Regulation. (2020c). *Impact of COVID-19 on consumer use and perception of telecommunication services*. ComReg Document 20/61. Dublin: Commission for Communications Regulation.

[CR11] Central Statistics Office (CSO). (2020). Census 2016 small area population statistics. https://www.cso.ie/en/census/census2016reports/census2016smallareapopulationstatistics/. Accessed 28 July 2020.

[CR12] Cullinan, J., & Duggan, J. (2016). A school-level gravity model of student migration flows to higher education institutions. *Spatial Economic Analysis,**11*(3), 294–314

[CR13] Cullinan, J. & Halpin, B. (2017). A Spatial Economic Perspective on Higher Education Choices. In J. Cullinan & D. Flannery (Eds.) *Economic insights on higher education policy in Ireland: Evidence from a public system*. London: Palgrave Macmillan.

[CR14] Davies, A. (2020). Australian universities take tentative steps towards opening after COVID-19. https://www.theguardian.com/australia-news/2020/jun/12/australian-universities-take-tentative-steps-towards-opening-after-covid-19. Accessed 15 July 2020.

[CR15] Department of Communications, Climate Action and the Environment. (2019). *Delivering the national broadband plan*. Dublin: Department of Communications, Climate Action & Environment.

[CR16] Department of Education and Skills. (2020). Minister Harris announces 17,000 laptops ordered to assist students with online and blended learning [Press release] 20 August. https://www.gov.ie/en/press-release/7143d-minister-harris-announces-17000-laptops-ordered-to-assist-students-with-online-and-blended-learning/. Accessed 21 August 2020.

[CR17] Dettling, L. J., Goodman, S., & Smith, J. (2018). Every little bit counts: the impact of high-speed internet on the transition to college. *Review of Economics and Statistics,**100*(2), 260–273

[CR18] Farrell, O., & Brunton, J. (2020). A balancing act: A Window into online student engagement experiences. *International Journal of Educational Technology in Higher Education,**17*, 25

[CR19] Federal Communications Commission. (2018). Broadband deployment plan [press release] 2 February. https://www.fcc.gov/reports-research/reports/broadband-progress-reports/2018-broadband-deployment-report. Accessed 29 July 2020.

[CR20] Figlio, D., Rush, M., & Yin, L. (2013). Is it live or is it internet? Experimental estimates of the effects of online instruction on student learning. *Journal of Labor Economics,**31*(4), 763–784

[CR21] Financial Times. (2020). YouTube, Amazon and Netflix cut picture quality in Europe. https://www.ft.com/content/70333747-f180-4887-8a26-27ab6b230299. Accessed 22 January 2021.

[CR22] Flannery, D., & Cullinan, J. (2017). Economics and higher education policy. In: *Economic insights on higher education policy in Ireland* (pp. 3–24). Palgrave Macmillan.

[CR23] Gaebel, M. (2020). COVID-19 and digitally enhanced learning and teaching: New opportunities in challenging times*.* European Universities Association. 28 May. https://www.eua.eu/resources/expert-voices/178-covid-19-and-digitally-enhanced-learning-and-teaching-new-opportunities-in-challenging-times.html. Accessed 28 July 2020.

[CR24] Gonzales, A. L., McCrory Calarco, J., & Lynch, T. (2018). Technology problems and student achievement gaps: A validation and extension of the technology maintenance construct. *Communication Research,**47*(5), 750–770

[CR25] Haase, T., & Pratschke, J. (2017). The 2016 Pobal HP deprivation index. www.trutzhaase.eu. Accessed 14 June 2020.

[CR26] Higher Education Authority. (2015). *National plan for equity of access to higher education 2015–2019.* Dublin: Higher Education Authority.

[CR27] Higher Education Authority. (2019). *A spatial and socio-economic profile of higher education institutes in Ireland.* Dublin: Higher Education Authority.

[CR28] Higher Education Authority. (2020). *Key facts & figures: Higher education 2017/18*. Dublin: Higher Education Authority.

[CR29] Kahu, E. R. (2013). Framing student engagement in higher education. *Studies in Higher Education,**38*(5), 758–773

[CR30] Kahu, E. R., Picton, C., & Nelson, K. (2020). Pathways to engagement: A longitudinal study of the first-year student experience in the educational interface. *Higher Education,**79*, 657–673

[CR31] Lamb, S., Maire, Q., Doecke, E., Macklin, S., Noble, K., & Pilcher, S. (2020). *Impact of learning from home on educational outcomes for disadvantaged children*. Victoria University.

[CR32] Lee, K. (2017). Rethinking the accessibility of online higher education: A historical review. *The Internet and Higher Education,**33*, 15–23

[CR33] McCoy, S., & Byrne, D. (2017). Student retention in higher education. In J. Cullinan & D. Flannery (Eds.), *Economic insights on higher education policy in Ireland: Evidence from a public system.*Palgrave Macmillan.

[CR34] McGuire, P. (2020). *Third-level teaching plans for Autumn, June 9th.* https://www.irishtimes.com/news/education/third-level-teaching-plans-for-autumn-1.4269916. Accessed 17 July 2020.

[CR35] Mohan, G., McCoy, S., Carroll, E., Mihut, G., Lyons, S., & Mac Domhnaill, C. (2020). *Learning for All? Second-level Education in Ireland During COVID-19*. ESRI Survey and Statistical Report Series No. 92. Dublin: Economic and Social Research Institute.

[CR36] Montacute, R. & Holt-White, E. (2020). *COVID-19 and social mobility impact brief: University access and finance*. Sutton Trust Research Brief*,* Sutton Trust.

[CR37] Naffi, N., Davidson, A.-L., Snyder, D. M., Kaufman, R., Clark, R. E., Patino, A., Gbetoglo, E., Duponsel, N., Savoie, C., Beatty, B., Wallace, G., Fournel, I., & Ruby (2020). Disruption in and by centres for teaching and learning during the COVID-19 pandemic. Quebec, Canada: International Observatory on the Societal Impacts of AI and Digital Technology. https://www.docdroid.com/L0khasC/whitepaper-disruption-in-and-by-centres-for-teaching-and-learning-during-the-covid-19-pandemic-leading-the-future-of-higher-ed-21-08-2020-pdf. Accessed 18 Apr 2021.

[CR38] National Forum for the Enhancement of Teaching and Learning in Higher Education. (2020). *INDEx finding from student and staff who teach in higher education*. Dublin: National Forum for the Enhancement of Teaching and Learning in Higher Education.

[CR39] New York Times. (2020). Surging traffic is slowing down our internet. https://www.nytimes.com/2020/03/26/business/coronavirus-internet-traffic-speed.html. Accessed 22 January 2021.

[CR40] Office for Students. (2020). Digital poverty’ risks leaving students behind [Press release] 3 September. https://www.officeforstudents.org.uk/news-blog-and-events/press-and-media/digital-poverty-risks-leaving-students-behind/. Accessed 16 January 2021.

[CR41] Ortagus, J. C. (2017). From the periphery to prominence: An examination of the changing profile of online students in American higher education. *The Internet and Higher Education,**32*, 47–57

[CR42] Palcic, D., & Reeves, E. (2011). *Privatisation in Ireland: Lessons from a European economy*. Palgrave Macmillan.

[CR43] Panigrahi, R., Srivastava, P., & Sharma, D. (2018). Online learning: Adoption, continuance, and learning outcome—a review of literature. *International Journal of Information Management,**43*, 1–14

[CR44] Paulsen, J., & McCormick, A.C. (2020). Reassessing disparities in online learner student engagement in higher education. *Educational Researcher*, published online 13 January 2020. https://doi.org/10.3102/0013189X19898690.

[CR45] Quality and Qualifications Ireland. (2018). *Statutory quality assurance guidelines for providers of blended learning programmes*. Dublin: Quality and Qualifications Ireland.

[CR46] Raes, A., Detienne, L., Windey, I., & Depaepe, F. (2019). A systematic literature review on synchronous hybrid learning: Gaps identified. *Learning Environments Research*, published online 28 November 2019. https://doi.org/10.1007/s10984-019-09303-z.

[CR47] Rasheed, A. R., Kamsin, A., & Nor, A. A. (2020). Challenges in the online component of blended learning: A systematic review. *Computers & Education,**144*, 103701

[CR48] Sanchis-Guarner, R. Montalban, J., & Weinhardt, F. (2021). Home broadband and human capital formation. *CESIFO Working Papers*, 8846, January 2021. SSRN: https://ssrn.com/abstract=3772087.

[CR49] Silva, S., Badasyan, N., & Busby, M. (2018). Diversity and digital divide: Using the national broadband map to identify the non-adopters of broadband. *Telecommunications Policy,**42*(5), 361–373

[CR50] Skinner, B. T. (2019). Making the connection: Broadband access and online course enrolment at public open admissions institutions. *Research in Higher Education,**60*(7), 960–999

[CR51] Staff, C. (2020). Here's a list of colleges' plans for reopening in the fall. Chronicle of higher education. https://www.chronicle.com/article/Here-sa-List-of-Colleges-/248626. Accessed 28 July 2020.

[CR52] Stich, A. E., & Reeves, T. D. (2017). Massive open online courses and underserved students in the United States. *The Internet and Higher Education,**32*, 58–71

[CR53] Union of Students in Ireland. (2020). *National report on students and COVID-19*. Dublin: Union of Students in Ireland. https://usi.ie/wp-content/uploads/2020/07/COVID_RESEARCH_FINAL.pdf. Accessed 28 July 2020.

[CR54] Walsh, S., Cullinan, J., & Flannery, D. (2017). The impact of proposed higher education reforms on geographic accessibility to universities in Ireland. *Applied Spatial Analysis and Policy,**10*, 515–536

[CR55] Walsh, S., Flannery, D., & Cullinan, J. (2015). Geographic accessibility to higher education on the Island of Ireland. *Irish Educational Studies,**34*(1), 5–23

[CR56] Xu, D., & Jaggars, S. S. (2013). The impact of online learning on students’ course outcomes: Evidence from a large community and technical college system. *Economics of Education Review,**37*, 46–57

[CR57] Xu, D. & Jaggars, S.S. (2013b). Adaptability to online learning: Differences across types of students and academic subject areas. *Community College Research Center Working Paper No. 54*, Columbia University, New York.

[CR58] Zydney, J. M., McKimmy, P., Lindberg, R., & Schmit, M. (2019). Here or there instruction: Lessons learned in implementing innovative approaches to blended synchronous learning. *TechTrends,**63*(2), 123–132

