# Supplementary material for: The disconnected: COVID-19 and disparities in access to quality broadband for higher education students
Source: Int J Educ Technol High Educ. 2021 May 21;18(1):26. doi: 10.1186/s41239-021-00262-1 (PMC8137268; doi:10.1186/s41239-021-00262-1)
Supplement: Supplementary file 1 — Additional file 1. Additional HEI and enrolment information. [file 41239_2021_262_MOESM1_ESM.docx]

**Appendix A: Additional HEI and Enrolment Information**

**Figure A.1: Irish HEIs by type and student enrolments by county**

**
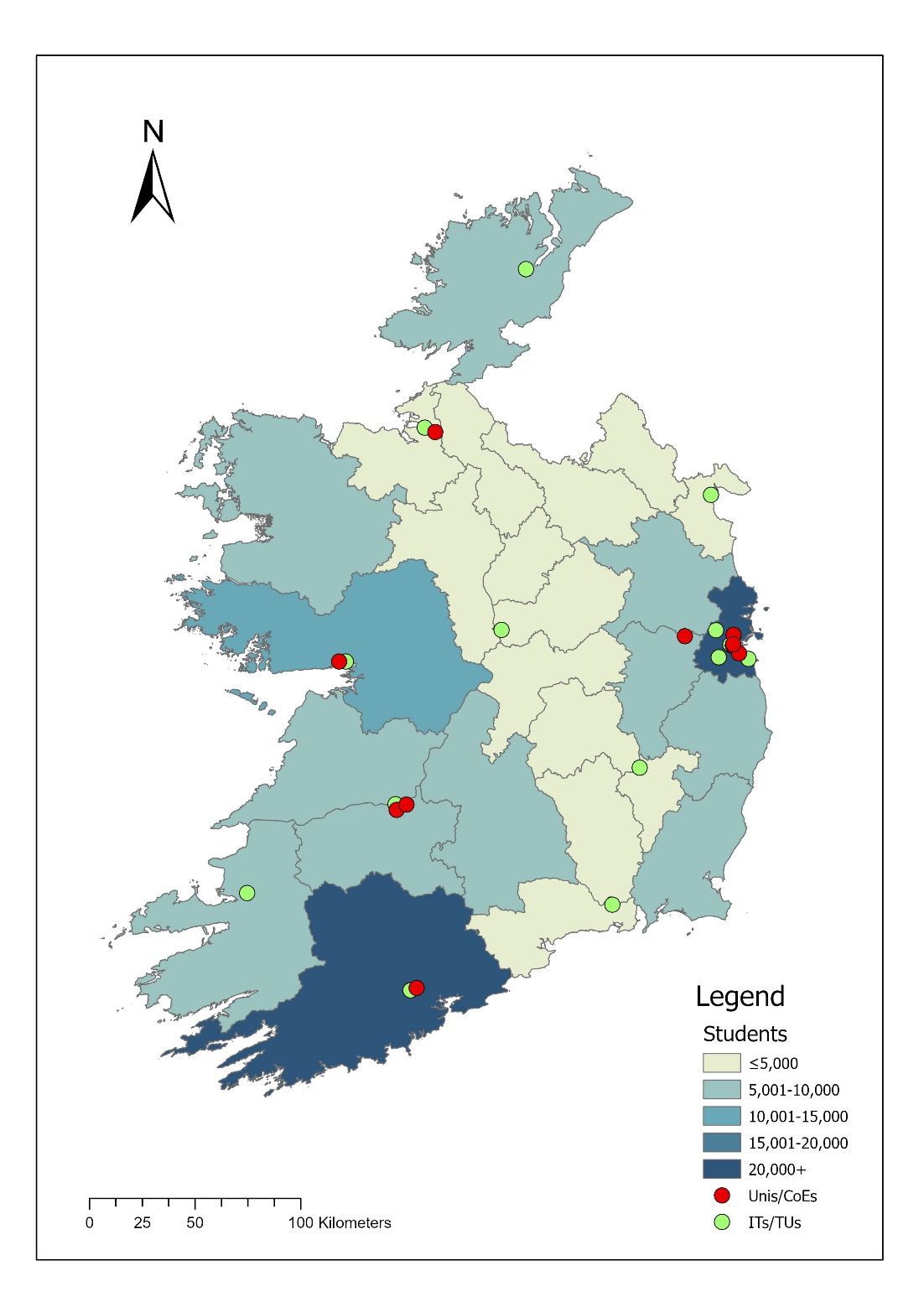
**

Source: Analysis of data from HEA.

**Figure A.2: Irish HEIs by size and student enrolments by county**


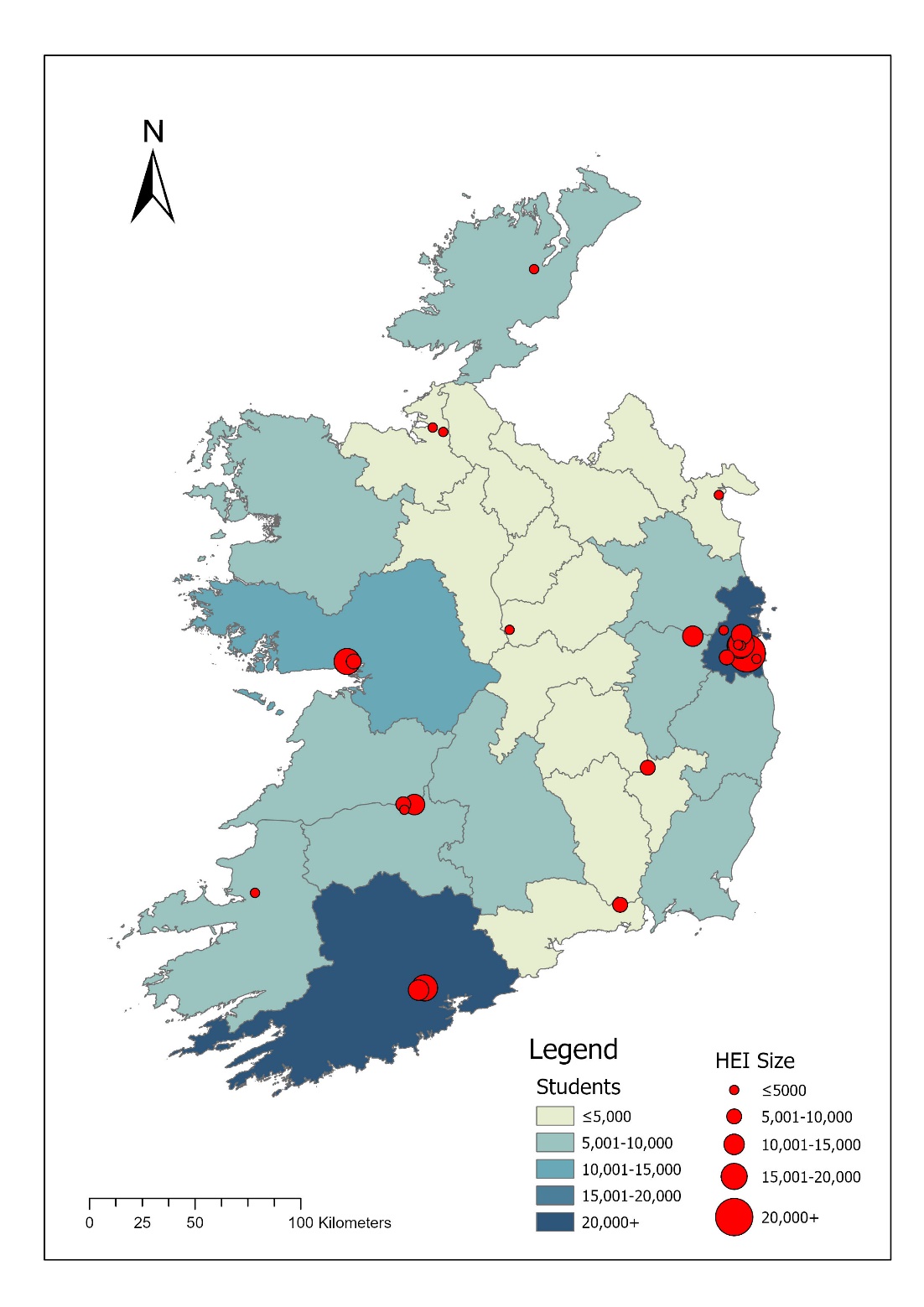


Source: Analysis of data from HEA.

**Table A.1: Enrolments by HEI (2017/18)**

| **HEI** | **Abbreviation** | **Enrolments** |
| --- | --- | --- |
| Athlone IT | AIT | 4,710 |
| Cork IT | CIT | 11,195 |
| Dublin City University | DCU | 15,704 |
| Dublin Institute of Technology | DIT | 18,508 |
| Dun Laoghaire Institute of Art, Design and Technology | IADT | 2,489 |
| Dundalk IT | DkIT | 4,898 |
| Galway-Mayo IT | GMIT | 6,581 |
| IT Blanchardstown | ITB | 2,905 |
| IT Carlow |  | 8,086 |
| IT Sligo |  | 3,693 |
| IT Tallaght |  | 5,204 |
| IT Tralee |  | 2,776 |
| Letterkenny IT | LyIT | 4,221 |
| Limerick IT | LIT | 6,077 |
| Mary Immaculate College, Limerick | Mary I | 4,771 |
| Maynooth University | MU | 12,287 |
| National College of Art and Design | NCAD | 1,145 |
| National University of Ireland Galway | NUIG | 18,365 |
| Royal College of Surgeons in Ireland | RCSI | 3,550 |
| St. Angela's College of Home Economics, Sligo | St. Angela's | 1,392 |
| University College Cork | UCC | 20,024 |
| Trinity College Dublin | TCD | 16,755 |
| University College Dublin | UCD | 26,508 |
| University of Limerick | UL | 14,120 |
| Waterford IT | WIT | 7,779 |
|  |  |  |
| Total |  | 223,743 |

Note: DIT, ITB and IT Tallaght amalgamated in January 2019 to become the Technological University of Dublin (TUD).

Source: HEA.

**Appendix B: Additional Broadband Subscription Information**

**Figure B.1: Broadband subscription proportions by platform, Q4 2018 – Q4 2019**


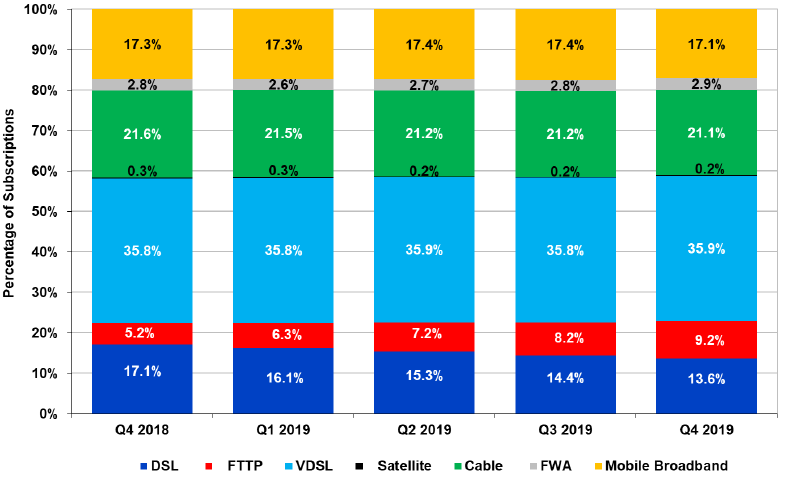


Source: ComReg (2020a).
